# Morphology of lithium halides in tetrahydrofuran from molecular dynamics with machine learning potentials†

**DOI:** 10.1039/d4sc04957h

**Published:** 2024-11-12

**Authors:** Marinella de Giovanetti, Sondre Hilmar Hopen Eliasson, Sigbjørn Løland Bore, Odile Eisenstein, Michele Cascella

**Affiliations:** a Department of Chemistry and Hylleraas Centre for Quantum Molecular Sciences, University of Oslo Oslo 0315 Norway michele.cascella@kjemi.uio.no; b ICGM, University of Montpellier, CNRS, ENSCM Montpellier 34293 France odile.eisenstein@umontpellier.fr

## Abstract

The preferred structures of lithium halides (LiX, with X = Cl, Br, I) in organic solvents have been the subject of a wide scientific debate, and a large variety of forms has been isolated and characterized by X-ray diffraction. The identified molecular scaffolds for LiX are diverse, often built on (LiX)_*n*_ rings with a prevalence of rhomboidal arrangements and an appropriate number of solvent or Lewis base molecules coordinating the lithium ions. Much less is known about the structures of LiX in solution, limiting the understanding of the synergistic role of LiX in reactions with various organometallic complexes, as prominently represented by the turbo Grignard reaction. Here, we trained a machine learning potential on *ab initio* data to explore the complex conformational landscape for systems comprising four LiX moieties in tetrahydrofuran (THF). For all the considered halogens a large number of scaffolds were found at thermally accessible free energy values, indicating that LiX in solution are a diverse ensemble constituted of (LiX)_*n*_ moieties of various sizes, completed by the appropriate number of coordinating THF. LiCl shows a preference for compact, pseudo-cubane Li_4_Cl_4_(THF)_4_ structures, coexisting with open rings. At concentrations close to the solubility limit, LiCl forms hexagonal structures, in analogy with literature observations on pre-nucleating NaCl. LiBr tends to favour less compact, more solvated aggregates. LiI significantly differs from the two other cases, producing highly solvated, monomeric, dimeric, or linear structures. This study provides a comprehensive view of LiX in organic solvent, revealing dynamical polymorphism that is not easily observable experimentally.

## Abstract 2

Using machine-learning potentials with *ab initio* accuracy, molecular dynamics simulations predict a diversity of structures for lithium halogen salts dissolved in tetrahydrofuran – from more compact LiCl, to more dispersed LiI.

## Graphical abstract

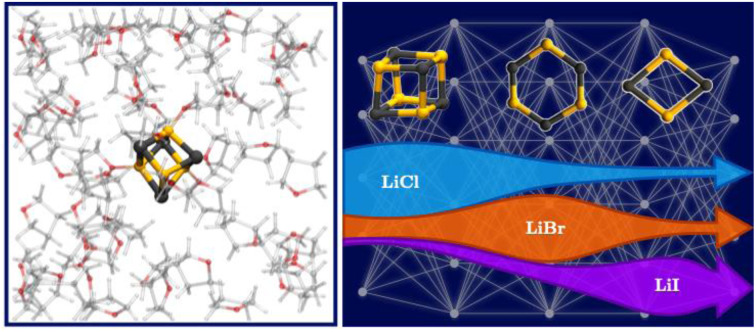

## 1 Introduction

Lithium halides (LiX) are important additives in organometallic chemistry, serving as pivotal agents that can modulate the reactivity and selectivity of organic reagents.^1^ The turbo Grignard reagent stands out as an emblematic example wherein the addition of lithium chloride empowers the performance of the organomagnesium species, enabling accelerated reaction rates, enhanced selectivity, and improved sensitivity.^2,3^ In fact, the literature indicates that LiX play a vital role in a multitude of chemical processes, establishing high-yield stereoselective aldol reactions,^4–9^ enhancing the reducing strength of inorganic salts,^10–13^ controlling polymer properties,^14–17^ and improving permeation and separation rates of polymeric membranes.^18–22^ Moreover, LiX constitute promising candidates for transition metal-free cathodes in batteries.^23,24^

**Fig. 1:**
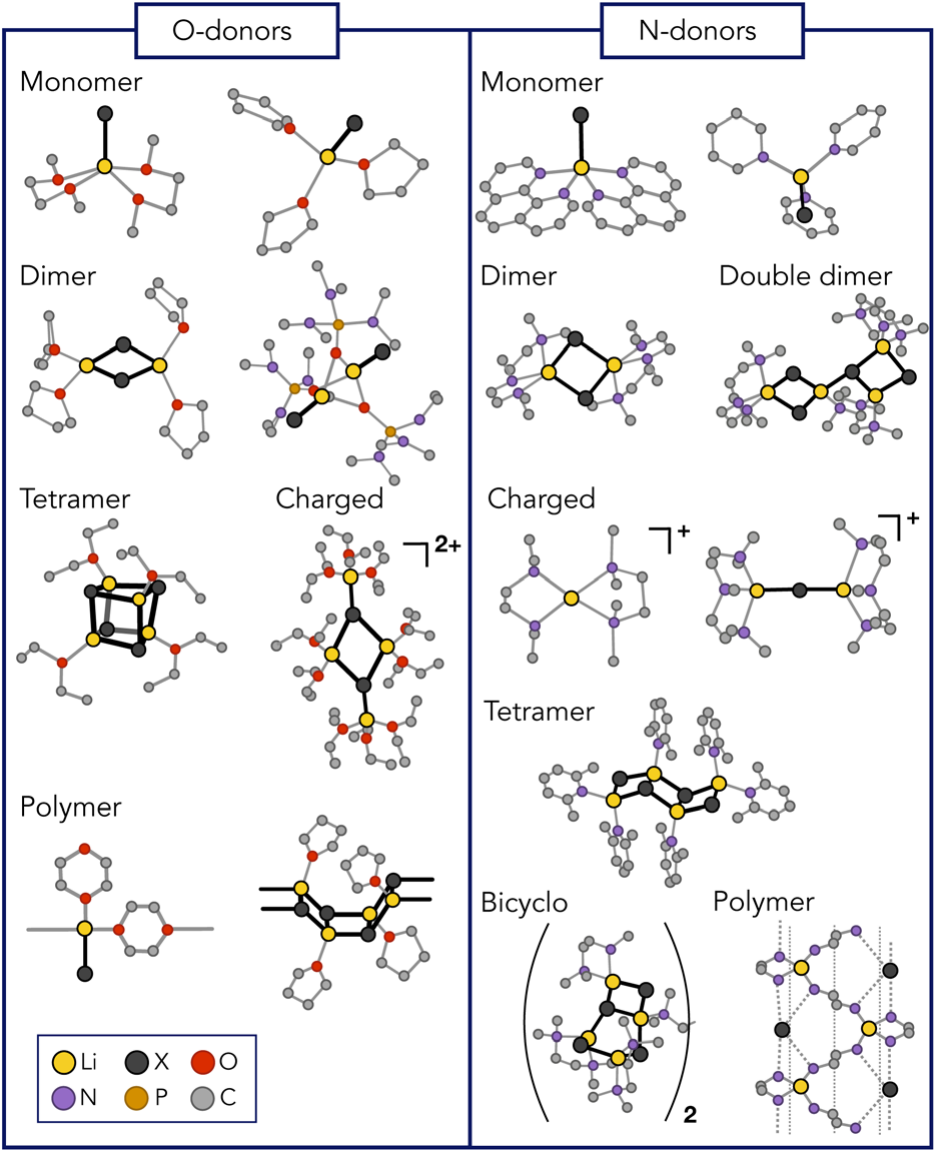
Lithium halide polymorphism. Representative conformations that were detected experimentally for different lithium halides with various O- and N-donor coordinating ligands.^25–51^ Hydrogens are not displayed for clarity.

As research in this field continues to evolve, and the synergistic interplay between LiX and organic reagents promises to unlock new pathways in chemical synthesis, it becomes essential to gain a comprehensive understanding of the chemistry of LiX. The task is challenging since numerous attempts to characterize LiX in organic solutions have evidenced a remarkable structural diversity. Depending on the conditions imposed, a diversity of moieties with varying nature and degrees of association can be expected.^25,26^ Accordingly, monomeric forms,^27–33^ as well as dimers,^28,30,33–38^ pseudo-cubanes,^25,39–41^ ladder-like structures,^30,42^ bridged forms,^43^ tetramers in double-dimeric arrangement,^28^ polymers,^44–49^ and ionic species have been detected (Fig. 1).^33,44,50^ It is therefore evident that LiX do not conform to a traditional identification as single, well-defined structures. Rather, they comprise an ensemble of morphologies that depend on the experimental conditions.

From this perspective, it is necessary to understand why such a multitude of species exists. Despite the considerable body of work on X-ray diffraction studies,^25–37,39–51^ there has been a paucity of investigations into the structural features of the species in solution, which represents the natural operating environment of these compounds. Furthermore, the results of these studies often differ. Lithium NMR spectroscopy experiments suggest that LiCl in tetrahydrofuran (THF) primarily exists as dimeric species, which can be deaggregated to monomeric entities by excess hexamethylphosphoramide.^52^ In contrast, LiBr would predominantly exist as a monomer,^52^ while LiI would form a contact ion pair, as well as a mixture of monomers and dimers.^52^ Another NMR study, also conducted in THF, proposed that LiX were to exist as ion-separated species regardless of the nature of the halide (X = Cl, Br, I).^53^ Finally, LiBr was reported to be a cubic tetramer in toluene.^54^ These studies were often conducted under low concentration and temperature conditions to minimize signal broadening due to dynamic exchange.^52^ Therefore, even from solution studies, the available information is often contradictory and does not reflect the conditions that these species experience under *operando* conditions. The aforementioned experimental challenges, coupled with the lack of information on high concentration, room temperature data, prompted our recent preliminary study of the structure of LiCl in THF^55^ with *ab initio* molecular dynamics (AIMD).^56^ This study underscored the existence of LiCl as a set of chemically distinct structures of high plasticity, in agreement with structures detected in experimental studies. The coexistence of species of different nature reflects stabilization effects from the solvation of the Li atoms, with ethereal solvent oxygens entering the coordination shell of lithium in competition with the halide. This is analogous to our precedent study on Grignard reagents, where solvent fluctuations were reported to be the driving force for the dimerization and disproportion dynamics, producing the different species involved in the Schlenk equilibrium.^57^ Our investigation also evidenced how the LiCl structure is prone to perturbation by Lewis acid/base interactions with organometallic species like Grignard derivatives. In particular, basic MgCl_2_ is capable of disintegrating the tetrameric pseudo-cubane Li_4_Cl_4_ cluster and forming species made of connected LiCl and Li_2_Cl_2_ with appropriate increased solvent coordination. This initial study thus established the existence of a vast conformational landscape dependent on the solvation state of the Li atoms. However, due to the high computational cost associated with AIMD simulations, a more complete characterization of the chemical space was unattainable, even when coupled with enhanced sampling approaches.

In recent years, machine learning potentials (MLPs) have become a popular solution for mitigating the computational cost associated with traditional *ab initio* methods.^58,59^ In this approach, MLPs, typically neural networks, are parameterized to predict forces of quantum calculations with speed-ups of two to three orders of magnitude. From pioneering MLPs based on invariant features, such as the Behler–Parrinello networks,^60^ or kernel-based approaches like the Gaussian Approximation Potential (GAP),^61^ to more recent equivariant graph neural networks, such as NequIP and MACE,^62,63^ MLPs have demonstrated their effectiveness in addressing a range of molecular problems. In particular, DeePMD is a conceptual evolution of the original network by Behler and Parrinello.^64,65^ It uses the local geometric environment to create a local descriptor followed by a feed-forward neural network to predict energies and forces from *ab initio* calculations.^64^ While DeepMD is one of the most computationally efficient MLPs available, it has also demonstrated an ability to reliably reproduce and predict the structural and dynamic properties of molecular systems in the condensed phase, including, but not limited to, the water phase diagram,^66–68^ combustion reactions,^69^ and reactions at interfaces.^70,71^ Electrolyte solutions have been studied for a long time by *ab initio* molecular dynamics,^57,72–81^ as well as by classical or polarizable force fields.^82–88^ More recently, MLPs have been effectively used to describe electrolyte systems,^89–92^ in particular molten salts,^93–97^ and to describe diffusion of ions in solid-state electrolytes.^98–101^ MLPs have also been recently used to investigate solvation effects, such as in works on solvation of ions^102^ and on formation of carbonate.^103^ MLPs have the advantage of incorporating many-body polarization effects with quantum mechanical accuracy, at much cheaper computational costs, and without the need of explicit parametrization and calibration over mechanical polarization models. In this work, we develop a MLP for LiX in THF (X = Cl, Br, I) to expand our preliminary study on LiCl^55^ to a more complete exploration of the possible structures of LiX in THF at room temperature.

## 2 Results

### 2.1 Validation of MLP

**Fig. 2:**
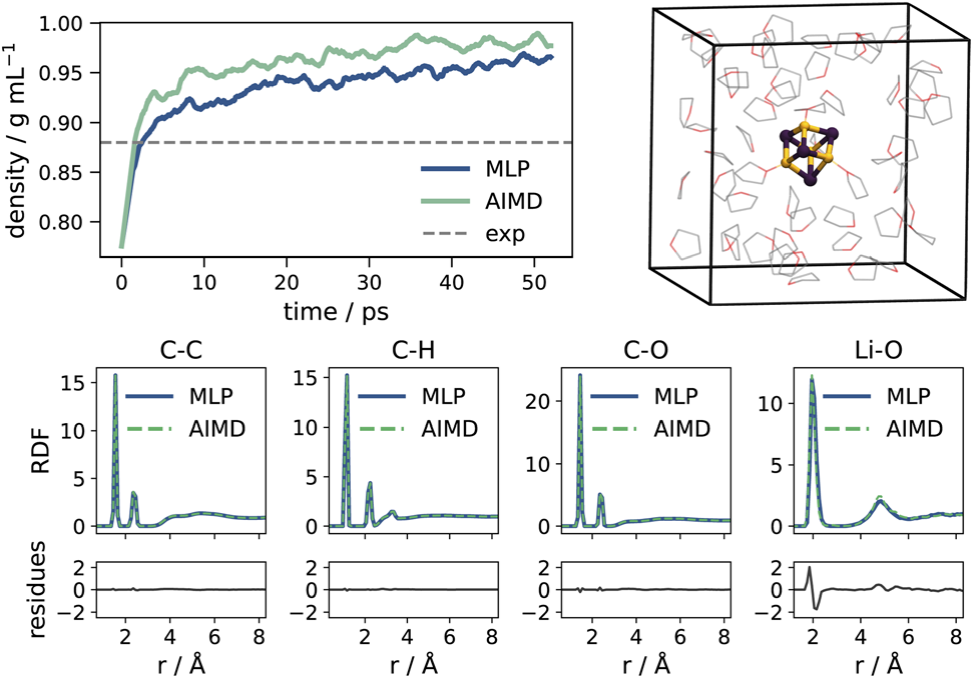
Validation of MLP. (Top, left) Density of a pure THF system. (Top, right) Simulation box for a sample Li_4_X_4_ system in THF; THF hydrogens omitted for clarity. (Bottom) Radial distribution functions (RDFs) over main atomic pairs.

The model is validated through a series of tests. First, we compute the density of pure THF at room temperature (Fig. 2). Relaxation of the isotropic pressure in the simulation box results in a density within 1% of the AIMD value.^104^ Next, we compute the radial distribution function (RDF) over main atomic pairs of a sample Li_4_Cl_4_ system in THF with the MLP, and compare it to the AIMD reference. As illustrated in Fig. 2, peak positions and probabilities are well reproduced across the bond pairs, with minimal residues. Only peak positions for Li–O interactions show a slight overestimation compared to the AIMD reference. Lastly, we verify that the MLP reproduces the AIMD Helmholtz free energy profiles as a function of the solvation of Li (as defined in eqn (S2)†) for the three Li_4_X_4_ systems with X = Cl, Br, I. The strategy and technical details for the development of the MLP are provided in the ESI.†

**Fig. 3:**
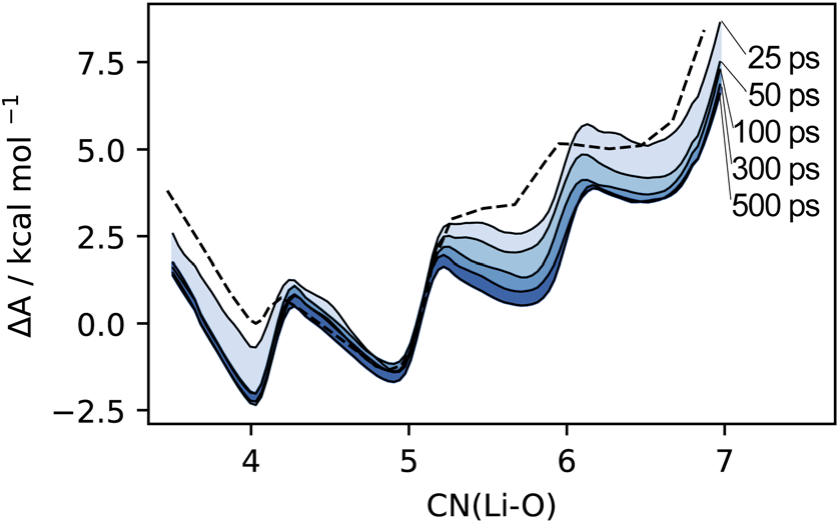
Time convergence of the Li_4_Cl_4_ Helmholtz free energy profile obtained with the MLP. The AIMD reference computed at timescales of 25 ps from ref. 55 is plotted as a dashed line.

Overall, the MLP profiles match well with the AIMD equivalents, and deviations mostly remain within the AIMD uncertainty. The profiles are reported in Fig. 3 and S5–S7.†

### 2.2 Lithium chloride

When sampled over a comparable timescale of 25 ps, the MLP closely follows the free energy profile of LiCl computed with AIMD,^55^ identifying weakly solvated, compact cubic structures as the most stable configurations (Fig. 3). These agree well with species C4 and C5 previously characterized by AIMD, with a slight lowering of the first minimum by about 0.5 kcal mol^−1^, and an associated increase in the barrier between the two species, although these differences fall within the uncertainty of the AIMD profile (Fig. S5†). As the simulation time increases, the free energy computed by the MLP becomes increasingly distinct from the AIMD profile in the region of higher solvation. In particular, the plateaus at CN(Li–O) = 6.0 and CN(Li–O) = 7.0 evolve into distinct local minima with an associated decrease in free energy. This behavior is governed by entropic effects, as longer simulations allow for a broader sampling of the conformational space and, thus, the identification of additional distinct species characterized by the same total number of coordinated THFs. The fact that this is observed only in the higher solvation region highlights how the morphology of LiCl can be effectively reduced to a combinatorial problem involving the total connectivity of the system. The possible arrangements of Li–Cl and Li–O(THF) links correlate with the number of THFs, leading to an increased number of possible structures for more solvated states. Because these species differ in the arrangement of Li–Cl and Li–O(THF) links, while retaining the same overall number of bonds, these species are essentially isoenergetic. Given the multitude of combinations, the restricted timescales accessible by AIMD limit access to statistically prevalent morphologies, with a notable bias towards the initial conformation. By employing an MLP, we reduce the computational burden of these simulations by over two orders of magnitude and enable a more comprehensive and unbiased sampling of the conformational space.

**Fig. 4:**
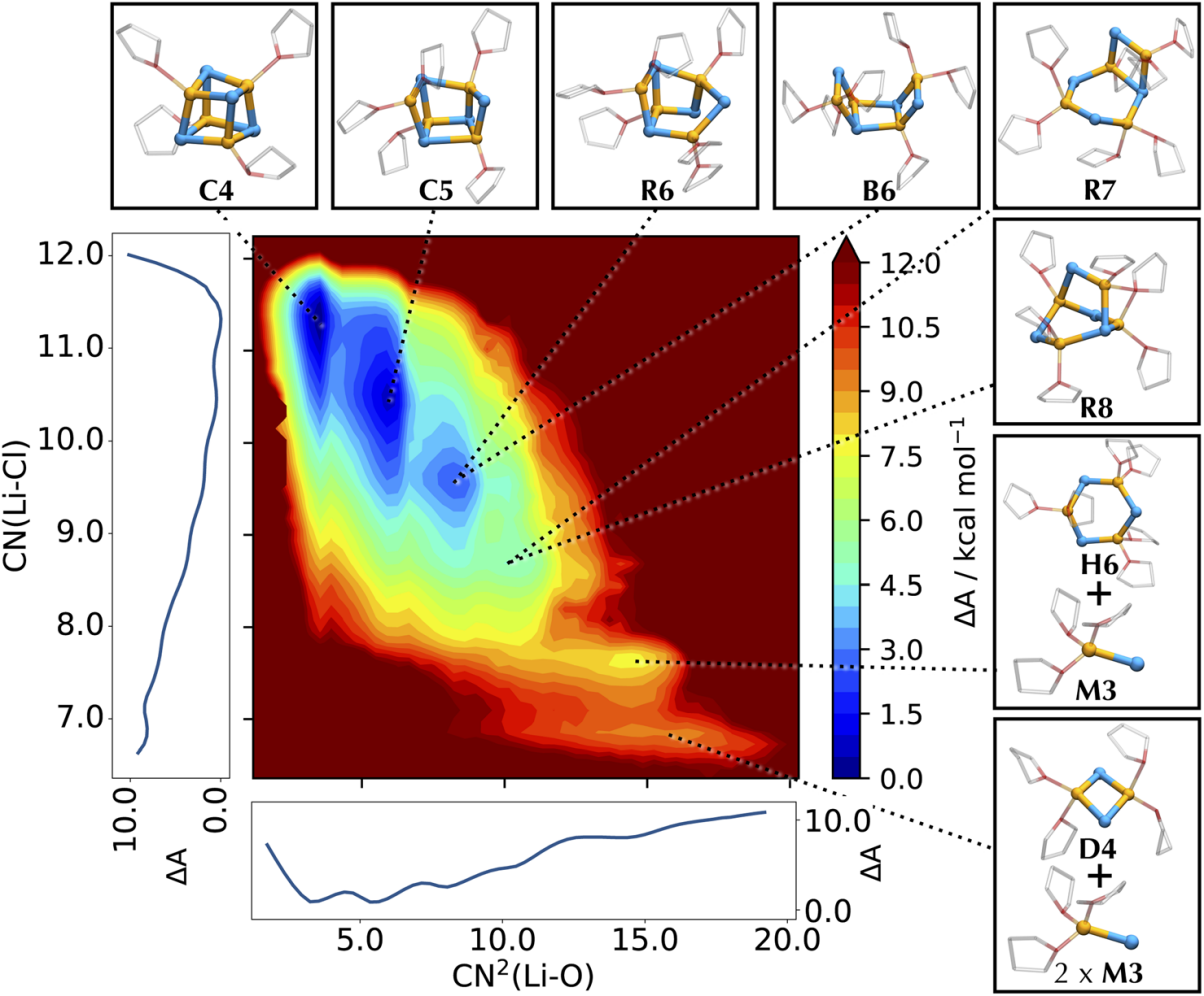
Helmholtz free energy surface of the conformational landscape of Li_4_Cl_4_. The coordination number of Cl around Li, CN(Li–Cl), and the squared coordination number of O around Li, CN^2^(Li–O), are used as collective variables for the FES. Mathematical definitions for the CVs are reported in ESI.† Side graphs report the projections of the FES onto the two respective axes. Representative structures corresponding to the basins of the FES are shown in balls-and-sticks (Li in yellow, Cl in cyan, and THF as transparent sticks).

To represent the different basins characterizing the system, we opt for a two-dimensional free energy surface (FES). As shown in Fig. 4, expanding the exploration of the conformational space in two dimensions permits the separation of different basins otherwise convoluted in the one-dimensional profile, allowing the characterization of both new and previously identified structures like C4, C5 and B6,^55^ and their assignment to well-defined basins of the FES. The majority of these structures retains the rhomboidal Li_2_Cl_2_ subunit, which is recognized as the fundamental unit of numerous LiX clusters.^36,40,41,45,47,49^ A second frequently encountered arrangement is a hexagonal ring of alternating Li and Cl (like H6), as also observed in some solid-state structures.^43^ The low solvation region is characterized by the two cubic structures C4 and C5, arising from the cleavage of one Li–Cl bond in C4, in analogy to the findings from the AIMD study.^55^ As the lithium solvation increases, the LiCl cubic cluster further degrades by cleaving a second Li–Cl bond into two possible species: a cyclic R6 structure (if breaking non-adjacent edges of adjacent faces), and a *cis* boat-type structure B6 (if breaking opposite edges of the same face). No *trans* conformer of B6 type is detected, suggesting a preference for the *cis* isomer.

While R6 and B6 occur in equal proportions, we note that R6 may be stabilized by the interaction with other compounds, as already noted for the interaction of Li_4_Cl_4_ with MgCl_2_.^55^ Further decomposition of the LiCl cluster leads to an ensemble of bicyclic and bridged structures with different THF coordination states, namely R7 and R8 and in a 8 : 1 ratio. These bicyclic species resemble LiCl structures observed in some crystal structures.^43^ Further down the solvation pathway, the degraded tetramer dissociates into a hexagonal and a monomeric unit, subsequently releasing another monomer into a mixture of lower molecular weight species. This corresponds to regions about 8–10 kcal mol^−1^ higher in energy, confirming the preference of LiCl for tetrameric clusters in solution compared to smaller aggregates.

### 2.3 Higher weight aggregates

**Fig. 5:**
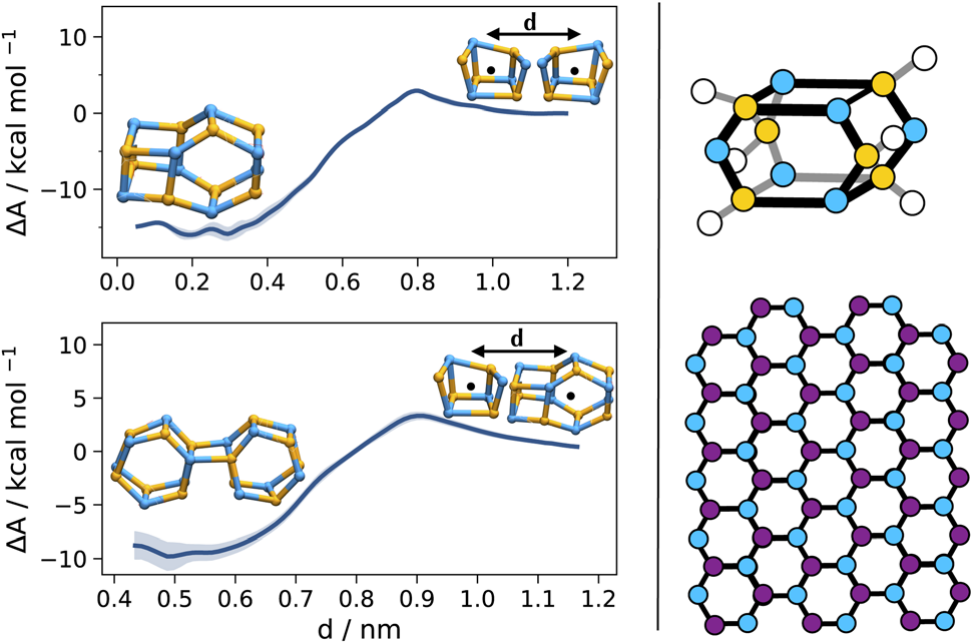
High aggregation states of LiCl. Computed binding affinities (left) and reported structures in the literature (right): hexagonal LiCl unit cells in polymeric solvent-separated crystals^33^ (right, top) and wurtzite-like arrangement in pre-nucleating NaCl precipitates^106^ (right, bottom). The free energy profiles are a function of the distance between the centres of mass of the two binding units (Li in yellow, Cl in cyan, and Na in purple; coordinating THF in the computed aggregates omitted for clarity). The shaded regions indicate 95% confidence intervals of estimated free energy profiles.

Given the preference for compact species (C4, C5), or for species that may condense into polymeric moieties (like B6),^30,42,45,47,48^ and given that LiCl is often used at a quasi-saturated concentration (1 M *versus* an experimental solubility of ≈1.27 M),^105^ we further investigate the likelihood of the formation of heavier aggregates. To maintain a concentration of 1 M, we double the simulation box, allowing for the presence of multiple Li_4_Cl_4_ tetrameric units that can interact with each other (second entry, Table S2†). The association of two tetrameric structures into an aggregate of 8 Li and 8 Cl nuclei requires an energy of 3.1(2) kcal mol^−1^ and leads to a stabilization of 16.1(8) kcal mol^−1^, while the addition of a third tetrameric structure has an energy barrier of 3.4(3) kcal mol^−1^ and leads to a further stabilization of −10(1) kcal mol^−1^. These two larger aggregates display units in hexagonal packing, as previously observed in polymeric solvent-separated crystals of LiCl in tetramethylenediamine.^33^ Low molecular weight aggregates in hexagonal packing have also been shown to form in the early stages of NaCl crystallization, preceding rock salt (FCC) formation (Fig. 5).^106^ These features suggest an initial nucleation stage of LiCl at a concentration close to, but still lower than, the saturation point. At the same time, quantum chemical calculations yield energies consistent with those predicted by the MLP (Table S3†). While this confirms that the MLP is able to reproduce data of the underlying quantum chemical quality, it also means that any shortcoming in the same quantum data used for training is inherited by the MLP. For larger aggregates containing several halide nuclei, strong dispersion forces are hard to reproduce accurately with density functional theory, even with dispersion corrections.^107^

### 2.4 Lithium bromide

**Fig. 6:**
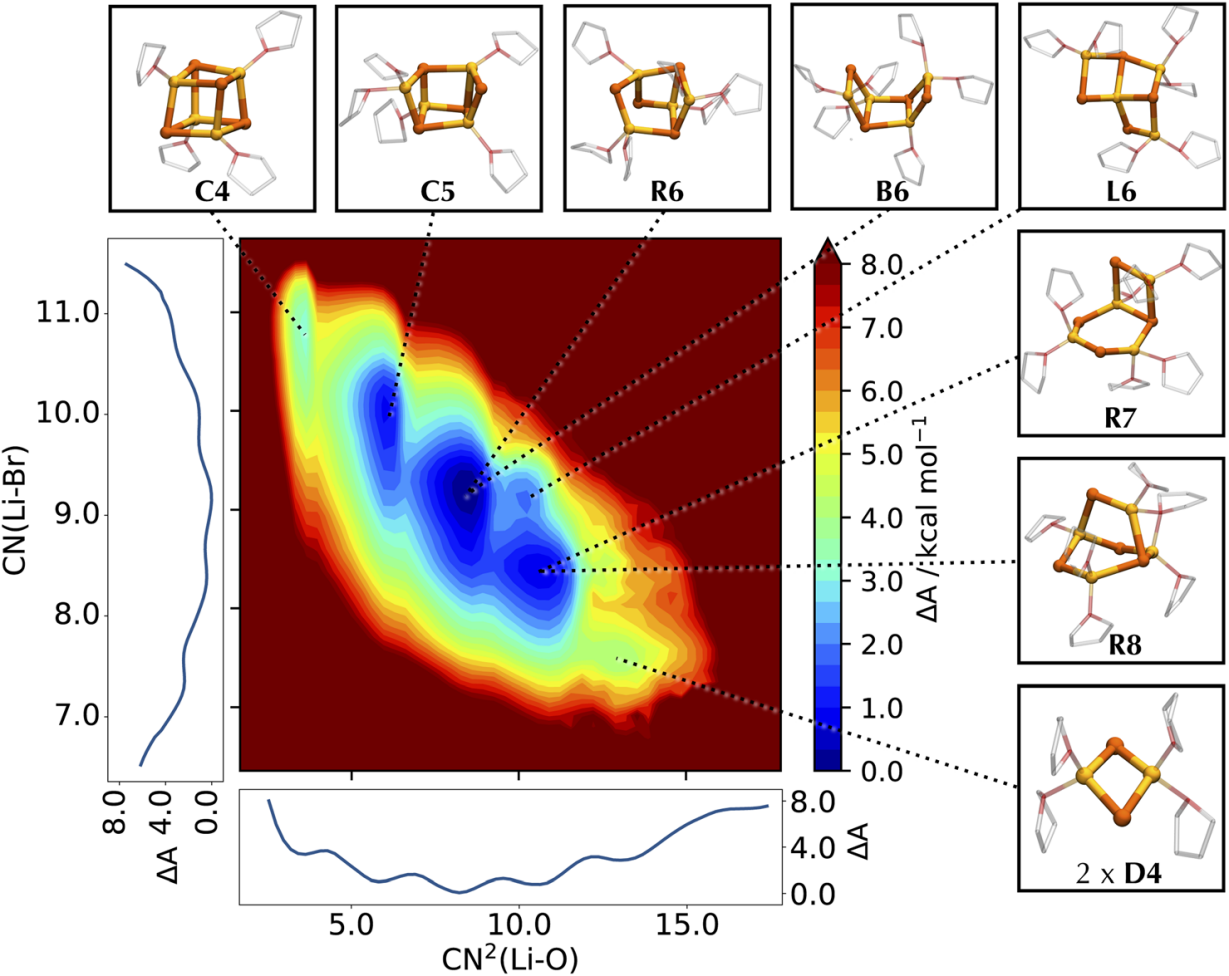
Helmholtz free energy surface of the conformational landscape of Li_4_Br_4_. The coordination number of Br around Li, CN(Li–Br), and the squared coordination number of O around Li, CN^2^(Li–O), are used as collective variables for the FES. Mathematical definitions for the CVs are reported in ESI.† Side graphs report the projections of the FES onto the two respective axes. Representative structures corresponding to the basins of the FES are shown in balls-and-sticks (Li in yellow, Br in orange, and THF as transparent sticks).

The free energy surface of Li_4_Br_4_ shows structures analogous to those obtained for Li_4_Cl_4_ with a preference for more solvated species (Fig. 6). The tetrasolvated pseudo-cubane C4 appears as a rather shallow metastable state, and there is a clear thermodynamic preference for an open form of the cube, C5, with a broken Li–Br bond and an additional THF at that lithium. Analogously to LiCl, C5 further degrades through the cleavage of a second Li–Br bond, resulting in cyclic R6 and a *cis* boat-like structure B6 in a 6 : 1 ratio. As in the case of LiCl,^47^ the structural arrangement in B6 corresponds to the core unit for THF-solvated crystalline LiBr polymers.^45^ Further down the solvation pathway, the FES of Li_4_Br_4_ exhibits a greater propensity for cyclic and bridged structures compared to LiCl. The obtained species correspond to the bromide analogues of bicyclic [4.2.0] R7 and bridged R8 in a 1 : 1 ratio. Increasing solvation further fragments the LiBr aggregate. Dimers of the type Li_2_Br_2_(THF)_4_ are slightly less stable (by 4 kcal mol^−1^) than any tetrameric species, confirming a preference for larger aggregates in solution. We do not detect any monomer across the free energy landscape, which are expected to occur at energies over 8 kcal mol^−1^.

### 2.5 Lithium iodide

**Fig. 7:**
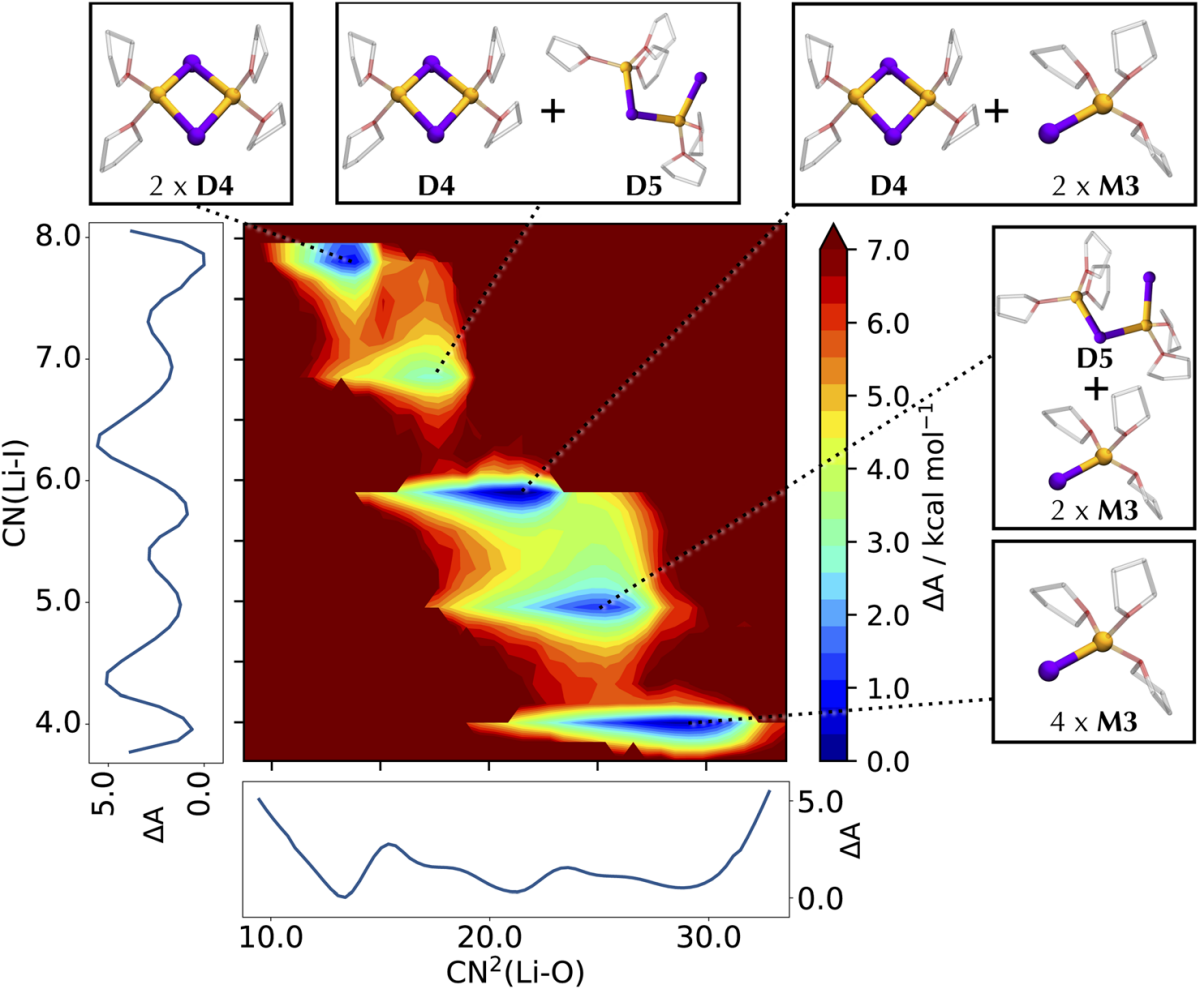
Helmholtz free energy surface of the conformational landscape of Li_4_I_4_. The coordination number of I around Li, CN(Li–I), and the squared coordination number of O around Li, CN^2^(Li–O), are used as collective variables for the FES. Mathematical definitions for the CVs are reported in ESI.† Side graphs report the projections of the FES onto the two respective axes. Representative structures corresponding to the basins of the FES are shown in balls-and-sticks (Li in yellow, I in purple, and THF as transparent sticks).

Unlike LiCl and LiBr, where dimeric units are notably thermodynamically disfavored compared to the tetrameric species, the free energy landscape of Li_4_I_4_ leads to the spontaneous degradation of the tetramer into smaller moieties (Fig. 7).

In our simulations, a starting pseudo-cubane type C4 structure promptly breaks down to form species of lower molecular weight through the transient formation of non-bridging moieties. Thus, the characterization of a tetrameric Li_4_I_4_(OEt_2_)_8_^27,39^ out of diethyl ether is an example of the difference in structural preference in the crystalline state and in solution. Such non-bridging moieties are observed to initiate the separation into dimers D4 already along the one-dimensional profile at the short AIMD timescales, indicating a preference for smaller aggregates (Fig. S7†). While dimeric moieties can also exist in a more solvated form (D5), where one Li–I bond is cleaved to accommodate an additional THF at the lithium, the closed-ring dimers remain more favored. Various mixtures of dimers and monomers are found further down the FES, where the fragmentation into monomeric species occurs beyond a barrier of 7 kcal mol^−1^.

### 2.6 Discussion

**Fig. 8:**
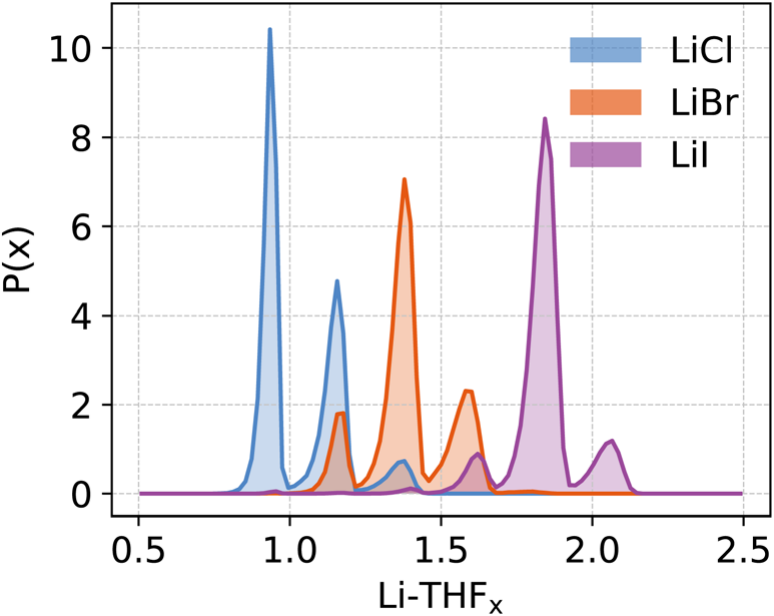
Probability distribution function (*P*(*x*)) of the stoichiometric ratio (*x*) of coordinating THFs at the Li centres for various lithium halides.

The distinct free energy landscapes of the lithium halides underscore the structural analogies and disparities between the various halides. In general, computational models predict multiple structures to be accessible to Li_4_X_4_ regardless of the nature of the halide. As expected, increasing the solvation of Li_4_X_4_ weakens or cleaves Li-X bonds. The solvent enters the first coordination sphere of lithium, mostly forming standard THF-Li terminal bonds; but THF may also assume bridging positions between two accessible lithium atoms, leading to a very high diversity of structures. In all cases, solvent substitution tends to follow a dissociative mechanism, with an initial elongation of the Li-X bond followed by the coordination of a THF, even though long-living under-coordinated Li intermediates are not observed during MD simulations. The preference for compact species like the pseudo-cubane declines from Cl to Br to I. The fact that the crystallization of the pseudo-cubanes Li_4_X_4_(S)_4_ (S = solvent) have been characterized for the three halides by X-ray diffraction for S = Et_2_O solutions^25,39,40^ illustrates the insightful comment by D. Seyferth that aggregates found in the crystalline state may not survive in solution.^109^ Additionally, the observation of pseudo-cubanes in the solid state for all three halides suggests that their absence in solution, particularly for iodide, is not due to an intrinsic destabilization for the heaviest halide. Instead, there is a solvent- or dynamic-driven preference for more solvated and, thus, smaller aggregates. Another marginal difference between the calculated and observed structures is that the ladder-type structure was not identified as a possible structure in solution while it has been reported for [LiCl(pyridine)]_n_,^110^ Li_4_Br_4_(2,6-dimethylpyridine)_6_^42^ and [LiI(THF)]_*n*_.^48^ Calculations identify only the boat form B6 for X = Cl and Br, a structure also observed in the crystalline state for [LiCl(THF)]_*n*_^47^ and [LiBr(THF)]_n_.^45^ Numerous factors determine the diversity in the aggregate sizes. Since many structures are common for the three halide systems, a reasonable hypothesis is to consider that the variation of the entropy term does not depend significantly on X. Therefore, one can focus on the enthalpic contribution. One noticeable difference between the three Li-X species is that the Li^*δ*+^–X^*δ*−^ charge polarization decreases from Cl to Br to I as shown for any given structure in Table 1 and Fig. S8.† Furthermore, this charge polarization is larger in smaller aggregates and in more open species where fewer halides are bonded to a given lithium. In particular, there is a significantly larger charge polarization in the LiX monomer relative to the Li_2_X_2_ dimer. Cleaving an Li-X bond is systematically associated with coordinating an additional THF to Li. Thus, the replacement of a Li⋯X interaction by a Li-THF coordination is likely to be energetically more favorable when the Li-X ionic character is minimized because the Li-X and Li-(THF) bond characters would be more similar. This favors the cleavage of Li-X and the increased solvation for heavier halides, as reported by the different solvation of the three halides salts (Fig. 8). It should be kept in mind that the solvent also has a stabilizing influence on the halide, which increases from Cl to Br to I. The dispersion interaction energy between THF and X is evaluated in the case of monomeric Li-X, yielding −7(3), −14(3), −18(4) kcal mol^−1^ for X = Cl, Br, I, respectively (Fig. S9 and Table S5†). Dispersion interaction has no preferential directionality and its increase is mostly due to the higher polarizability of the heavier halides. The outcome of these effects is that a LiX aggregate with greater ionic character within the Li-X bond tends to prefer polynuclear forms with numerous Li⋯X interactions. In contrast, with heavier X and an associated less ionic character of the Li-X bond, the Li⋯X interaction is more in competition with Li⋯THF coordination supplemented by X⋯THF interplays, and the fragmentation of the polynuclear aggregates into smaller units becomes more favorable. However, this fragmentation reaches a limit since the Li-X ionic character is higher in smaller units. This is why the monomer is not found on the free energy maps as an accessible species, on its own, within the energy range considered in the study, the exception being for X = I where it occurs at higher energy (Δ*A* ≈ 5 kcal mol^−1^) and thus in a relatively smaller concentration. For the other halides, the dimeric species is the smallest aggregate identified as energetically accessible. Finally, it is interesting to remember that the solubility of LiX in organic solvents increases from Cl to Br to I.^111,112^ While this study does not address the topic of solubility of these ionic salts, the parallel between our results and the trend in solubility is enthralling. Certainly, the favored fragmentation of large aggregates participates in the solubilization of LiX.

**Table 1:** Halogen atomic charges, computed with the CM5 method,^108^ for different Li coordination spheres in LiX salts (Li in yellow, X in black, and THF as transparent sticks)

	μ_1_-Li	μ_2_-Li	μ_3_-Li
	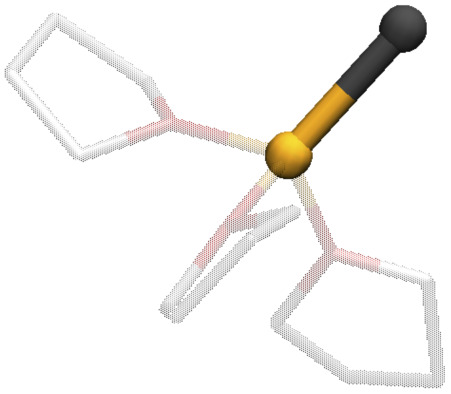	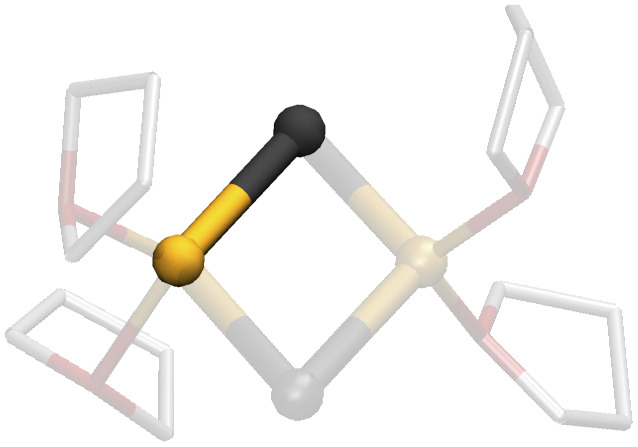	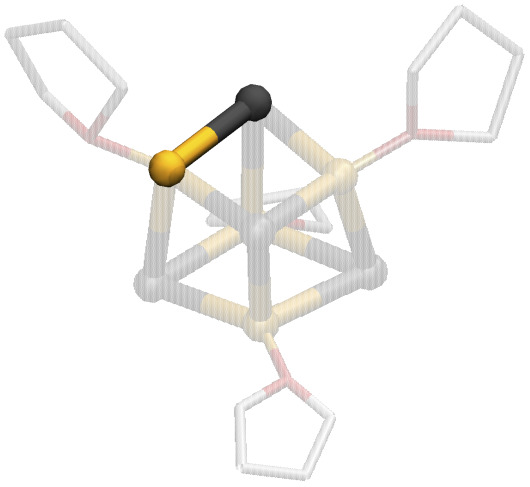
Cl	−0.633	−0.491	−0.425
Br	−0.608	−0.453	−0.372
I	−0.593	−0.425	−0.302

The absolute bond lengths and the expected slight shortening of Li-X bond distances upon opening the pseudo-cubane seen experimentally for X = Cl are satisfactorily reproduced. The Li–Cl distance in Li_2_Cl_2_(THF)_4_ was reported to be 2.308(3) and 2.342(3) Å.^36^ In the pseudo-cubane structure, the Li–Cl bond distance was also estimated to be between 2.357(10) and 2.441(12) Å.^41^ This agrees well with calculated Li–Cl distances of 2.3(2) and 2.4(2) Å for the dimer and tetramer, respectively. The Li–Br bond distance was found to be around 2.53 Å in the broken cube polymer [(LiBr)(THF)]_*n*_,^45^ and was mentioned to be slightly longer in different tetrameric species,^25,42^ fitting well a value of 2.6(4) Å in our simulations. In the dimeric form, the computed distances of 2.5(2) Å are consistent with reported values of 2.51(2)–2.55(2) Å.^113^ For Li–I, distances of 2.741(7) Å in the monomer^27^ and between 2.787(7) and 2.825(7) Å in the cubane^39^ are also in good agreement with our data (2.7(2) and 2.8(2) Å, respectively).

The calculations reported here suggest that lithium halides are present in solution in a wide number of species. Their shape and relative abundances are strongly determined by the solvent, even if the nature of X tends to direct the systems towards compact species (X = Cl) or small and open species (X = I). Other solvents, like diethyl ether, are likely to yield similar global energy patterns, but different in their quantitative details. Adding other salts can also have drastic effects, as indicated in our early finding that MgCl_2_ dismantles a pseudo-cubane Li_4_Cl_4_ into a globally open structure.^55^ Thus, these lithium salts have geometrical features that are almost entirely determined by their environment.

## 3 Conclusions

This study reveals the considerable variety of structural forms that are energetically accessible for lithium halides (LiX, with X = Cl, Br, I) in THF solution. This profusion is due to the intricate pattern governed by the flexibility of the coordination sphere of lithium, the solvent dynamics, and the competition between intramolecular and intermolecular interactions. The wide exploration of the chemical space was made possible only by using an MLP trained on a comprehensive set of AIMD data. In particular, the MLP enabled the investigation of extended timescales with remarkable computational efficiency. These extended time scales were essential in the discovery of possible highly solvated and flexible species. Exploration of the chemical space revealed several key findings. The LiCl species were found to have the largest variety of forms. This free energy surface is essentially flat, containing the emblematic relatively poorly solvated pseudo-cubane and diversely less compact and more solvated species made generally of connected rings of size often larger than four. Additionally, high-weight aggregates of LiCl were studied, where calculations revealed the preference for connected hexagonal units resulting from merging several pseudo-cubanes. The agreement with available experimental structures supports our methodological approach for modeling lithium chloride in solution. Similar calculations for lithium bromide and lithium iodide also showed a great diversity of structures, with a clear tendency to prefer less compact, smaller and more solvated LiX aggregates in which Li⋯X intramolecular interactions are replaced by THF coordination. The structural preferences are proposed to be related to the ionicity of the Li-X bond, which is qualitatively described by the atomic charges. The ionic character decreases from Cl to Br to I, and increases as the size of the LiX aggregates shrinks, with a substantial rise from dimer to monomer. As it is easier for THF to cleave a less ionic Li-X bond, this accounts well for the observed trends. It also accounts for the fact that monomeric LiX is not found to be energetically accessible except for I.

The key result of this study is to establish that a solution of LiX in THF is an ensemble of numerous structures coexisting in relatively similar concentrations. Fishing out a given species by adding a given Lewis base is just picking out the species that fit the additive. However, validating its presence does not address the other aggregates that also exist. There are in fact many fishes made from LiX swimming together in a pool of THF.

## Data availability

Data supporting this article are available in the ESI.† Simulation data presented in this work are openly available at the GitHub repository https://github.com/Cascella-Group-UiO/Publications.

## Author contributions

M. C. and O. E. conceptualized the project. S. L. B. wrote the code that automatized active learning iterations for MLPs used in this work. M. d. G., S. L. B., and S. H. H. E. calibrated the machine learning potential. M. d. G. ran all the simulations, analyzed the data, and wrote the first draft of the manuscript. All authors contributed to writing and reviewing the final version of the manuscript.

## Conflicts of interest

There are no conflicts to declare.

## Supplementary Material

SC-OLF-D4SC04957H-s001
