# Supplementary material for: Morphology of lithium halides in tetrahydrofuran from molecular dynamics with machine learning potentials
Source: Chem Sci. 2024 Nov 12;15(48):20355–64. doi: 10.1039/d4sc04957h (PMC11577267; doi:10.1039/d4sc04957h)
Supplement: SC-015-D4SC04957H-s001 [file SC-015-D4SC04957H-s001.pdf]

Supplementary Information (SI) for Chemical Science.
This journal is © The Royal Society of Chemistry 2024

Supporting Information: Morphology of Lithium Halides in Tetrahydrofuran from Molecular Dynamics with Machine Learning Potentials

Marinella de Giovanetti,[†] Sondre H. Hopen Eliasson,[†] Sigbjørn Løland Bore,[†] Odile Eisenstein,[*,‡,†] and Michele Cascella[*,†]

†*Department of Chemistry and Hylleraas Centre for Quantum Molecular Sciences, University of Oslo, 0315 Oslo, Norway*

‡*ICGM, University of Montpellier, CNRS, ENSCM, 34293 Montpellier, France*

E-mail: odile.eisenstein@umontpellier.fr; michele.cascella@kjemi.uio.no

Contents

S1 Machine Learning potential **S2**

S1.1 Development of the model . . . S2

S1.2 Selection and labelling of configurations . . . S5

S1.3 Validation . . . S6

S1.4 Production runs . . . S9

S1.4.1 One-dimensional solvation pathways of Li_4X_4 . . . S9

S1.4.2 Higher LiCl aggregates . . . S10

S1.4.3 Two-dimensional free energy maps of Li_4X_4 . . . S11

S2 Quantum chemical energies of higher weight LiCl **S11**

S3 Atomic charges **S12**

S4 Probability distribution function for Li-THF **S14**

S5 Halogen-THF affinity **S15**

References **S16**

S1 Machine Learning potential

S1.1 Development of the model

The machine learning potential (MLP) applied in this study was developed with the smooth edition of the deep potential (DeepPot-SE),[1] as outlined in Ref.[2]. We employ a two-atom embedding descriptor, where atomic information is encoded through a hidden embedding net of three layers of 25, 50, and 100 neurons, respectively, while the submatrix of the embedding matrix employs 16 neurons. Neighbor information is encoded till a cutoff distance of 6.0 Å, with a smoothing region of 0.5 Å. We note that even though a 6.0 Å cutoff is used, the many-body potential effectively accounts for interactions up to 12 Å and infers, to some degree, long-range interactions into short-range ones. Each deep neural network (DNN) potential is characterized by a fully connected architecture featuring three layers comprising 240 neurons each. We opted to use hyperparameter settings as used in Ref.[3], getting a reasonable accuracy for forces, with no overfitting as indicated by consistent validation errors.

The model was developed through an active learning approach. An initial training pool consisting of ab initio molecular dynamics (AIMD) snapshots was systematically expanded with configurations gathered through molecular dynamics (MD) sampling with the MLPs. Configurations were sampled in the NPT ensemble, and further enrichment was achieved in the NVT ensemble. For the former, a Nosé-Hoover barostat[4] with time constant 1 ps and Nosé-Hoover thermostat with time constant 0.5 ps were employed. For NVT runs, we employed a canonical sampling through velocity rescaling (CSVR) thermostat[5] with relaxation time of 100 ps. As demonstrated in Ref.[6] and Ref.[7], we used enhanced sampling simulations to gather out-of-equilibrium configurations relevant to the reactions of the present study. Data was collected across various thermodynamic conditions and aggregation states (i.e., including monomeric, dimeric, and tetrameric forms of all three LiX salts). The training pool was enriched with structures of organolithium compounds, to increase stability and transferability of the potential. For LiCl, the concentrations varied in the range of 0.8 M to 1.6 M. A query-by-committee strategy was employed to select relevant configurations sampled in MD trajectories based on the standard deviations of the atomic forces between an ensemble of three independently trained models. We selected configurations for labelling in the interval between 0.2 and 2.0 eV $Å^{-1}$ (refer to next section for details). The force interval is selected to include a broad range of structures in

the training set, and consequently, to improve the accuracy and robustness of the model. The full training set is summarized in Table S1. This includes a total of 23917 configurations, divided into training and validation in a 0.95 : 0.05 ratio.

Table S1: Composition of the training set data and corresponding thermodynamic conditions.

System	Box side length	Thermodynamic range	Number of configurations
25 THFs	8-19 Å	200-2000 K, 1-100000 bar	1798
Li_4Cl_4 + 55 THFs	20.1 Å	200-500 K, 1-1000 bar	4875
Li_8Cl_8 + 53 THFs	20.1 Å	200-300 K, 1 bar	82
$Li_{40}Cl_{40}$ + 520 THFs	40.6 Å	300-350 K, 1 bar	88
$Li_{48}Cl_{48}$ + 424 THFs	38.1 Å	300 K, 1 bar	202
Li_4Cl_3Me + 55 THFs	20.1 Å	300 K, 1 bar	52
$Li_4Cl_2Me_2$ + 55 THFs	20.1 Å	300 K, 1 bar	226
Li_4ClMe_3 + 55 THFs	20.1 Å	300 K, 1 bar	30
$Li_8Cl_4Me_4$ + 55 THFs	20.1 Å	100-350 K, 1 bar	3731
Li_4Br_4 + 55 THFs	20.1 Å	200-300 K, 1 bar	6621
Li_8Br_8 + 55 THFs	20.1 Å	300 K, 1 bar	2
Li_4Br_3Me + 55 THFs	20.1 Å	300 K, 1 bar	106
$Li_4Br_2Me_2$ + 55 THFs	20.1 Å	300 K, 1 bar	338
Li_4BrMe_3 + 55 THFs	20.1 Å	300 K, 1 bar	150
Li_4I_4 + 55 THFs	20.1 Å	200-300 K, 1 bar	1428
Li_4I_3Me + 55 THFs	20.1 Å	300 K, 1 bar	105
$Li_4I_2Me_2$ + 55 THFs	20.1 Å	300 K, 1 bar	219
Li_4IMe_3 + 55 THFs	20.1 Å	300 K, 1 bar	54
Li_4Me_4 + 55 THFs	20.1 Å	200-350 K, 1 bar	3809
Li_4iPr_4 + 55 THFs	20.1 Å	300 K, 1 bar	1

The potential was trained for 3.0×10^5 steps with a learning rate decreasing exponentially from 0.001 to 3.5×10^{-8} every 5000 steps, and a batch size of 100. The initial weighting factors for the energy and force losses were set to 0.02 and 1000, respectively, and both linearly converged to 1.0 during training. The learning curve is presented in Figure S1, with a final training set error for energies and forces of 2.88×10^{-2} kcal mol^{-1} and 1.55 kcal mol^{-1}Å^{-1}, respectively. The final validation error for energies and forces are instead 4.15×10^{-3} kcal mol^{-1} and 1.81 kcal mol^{-1}Å^{-1}, respectively.

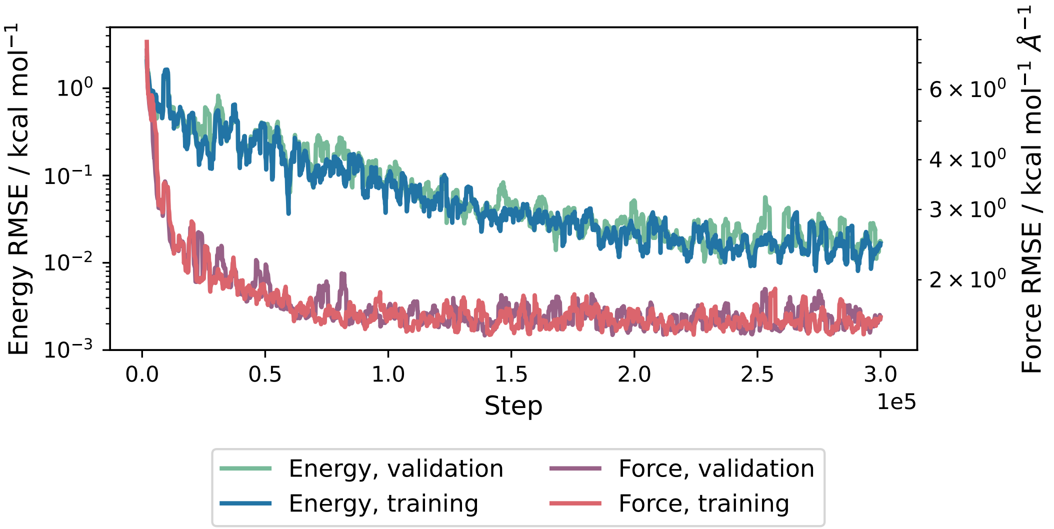

Figure S1: Learning curve for the final MLP model used to gather the results presented in this manuscript. The RMSEs plotted correspond to averages over 2000 training steps.

The root mean squared errors (RMSEs) on the energies and forces for the training set are given below:

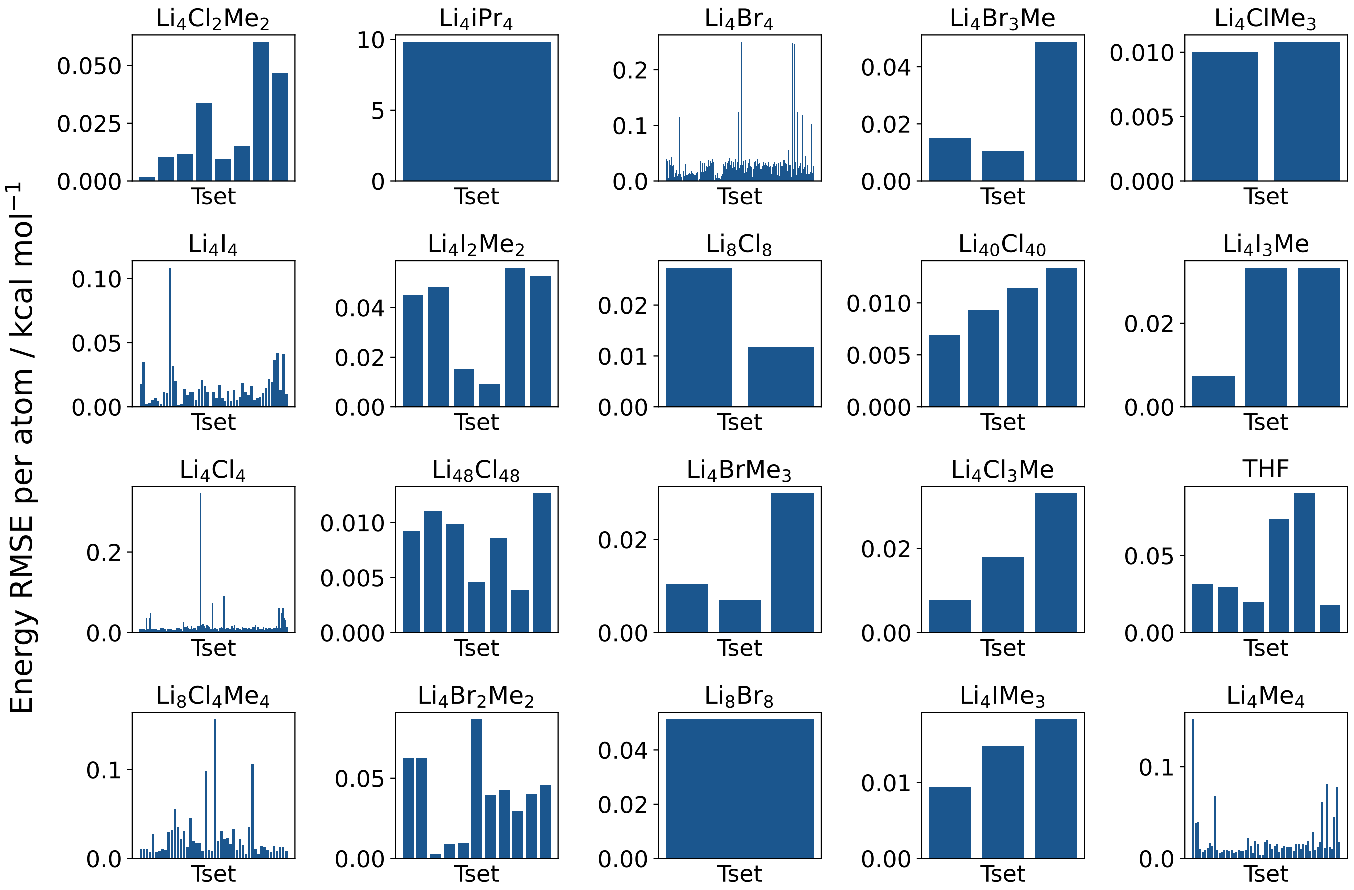

Figure S2: Energy RMSE per atom for the training set, shown for each system type. Plot headers give the simulated systems in THF, as listed in Table S1.

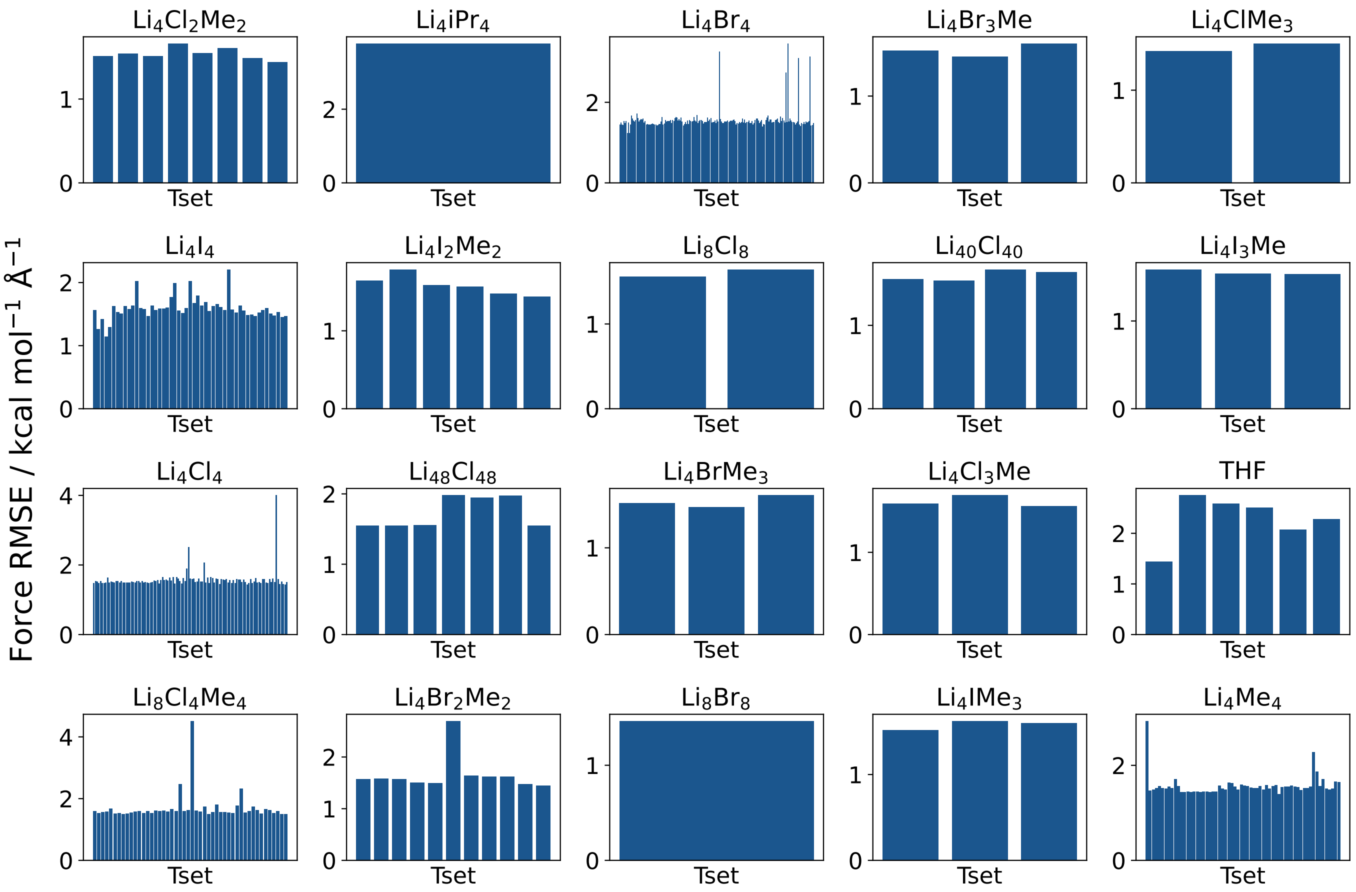

Figure S3: Force RMSEs for the training set, shown for each system type. Plot headers give the simulated systems in THF, as listed in Table S1.

S1.2 Selection and labelling of configurations

For the configurations iteratively selected and incorporated as training data, the atomic forces were calculated at the density functional theory (DFT)[8,9] level using the Perdew-Burke-Ernzerhof (PBE) exchange-correlation (xc) functional.[10,11] The Kohn-Sham orbitals were expanded over mixed Gaussian and plane-wave basis functions.[12] For the former, a DZVP basis set was used for all atoms in a molecular optimized formulation for halogen atoms.[13] The auxiliary plane-wave basis set was expanded up to an 800 Ry cutoff, which was determined sufficient to be consistent with state-of-the-art (SOTA) force accuracy of machine learning potentials (Figure S4). The integration of core electrons was achieved by utilizing pseudopotentials of the Goedecker-Teter-Hutter type (GTH).[14] Dispersion forces were accounted for though the D3 Grimme approximation.[15] AIMD forces and energies were computed over the ground-state potential energy surface, with a threshold for convergence of the

energy gradient of 1.0×10^{-5} au. Throughout such simulations, we employ the CP2K software.[16]

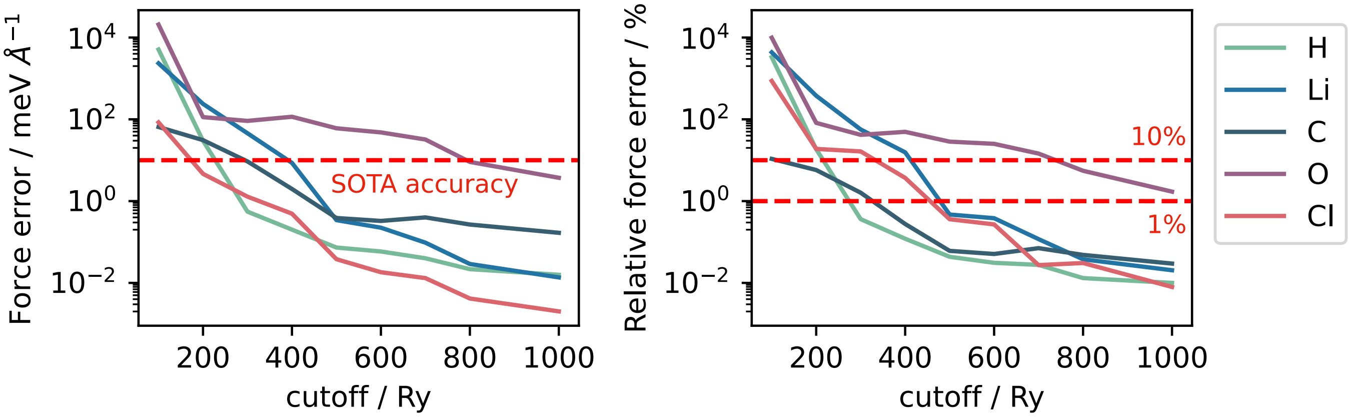

Figure S4: Benchmark of the auxiliary plane-wave basis set cutoff in quantum chemical calculations. Error analysis was conducted relative to a reference cutoff of 1500 Ry. Results obtained from a snapshot taken at 300 K and 1 bar during an NVT simulation of a Li_4Cl_4 system in THF.

S1.3 Validation

Validation tests are presented in the main text and include:

- The density of pure THF at 300 K and 1 bar, corresponding to system of entry 1, Table S1; the densities were computed in the NPT ensemble employing a Nose-Hoover barostat[4] with time constant 0.1 ps for both MLP and AIMD, and Nose-Hoover thermostat for MLP and CSVR[5] thermostat for AIMD, both of time constant 0.05 ps; plotted values correspond to averages over 4000 timesteps.

- The radial distribution functions (RDFs) over main atomic pairs, corresponding to system of entry 2, Table S1; the RDFs were computed in the NVT ensemble at 300 K, employing a CSVR thermostat[5] with a time constant 0.5 and 0.05 ps for MLP and AIMD, respectively;

- The free energy profiles of Li_4X_4 for X = Cl, Br, I along the solvation of the lithium (as defined in Equation S2); these correspond to systems of entries 2,10,15, Table S1; MLP profiles were estimated from umbrella sampling simulations[17] (see next section for details), while AIMD profiles were computed through thermodynamic integration of the constraint forces in the blue-moon ensemble[18]. The AIMD free energy profiles for Li_4Cl_4,[19] Li_4Br_4 and Li_4I_4 are shown in Figures S5, S6 and S7.

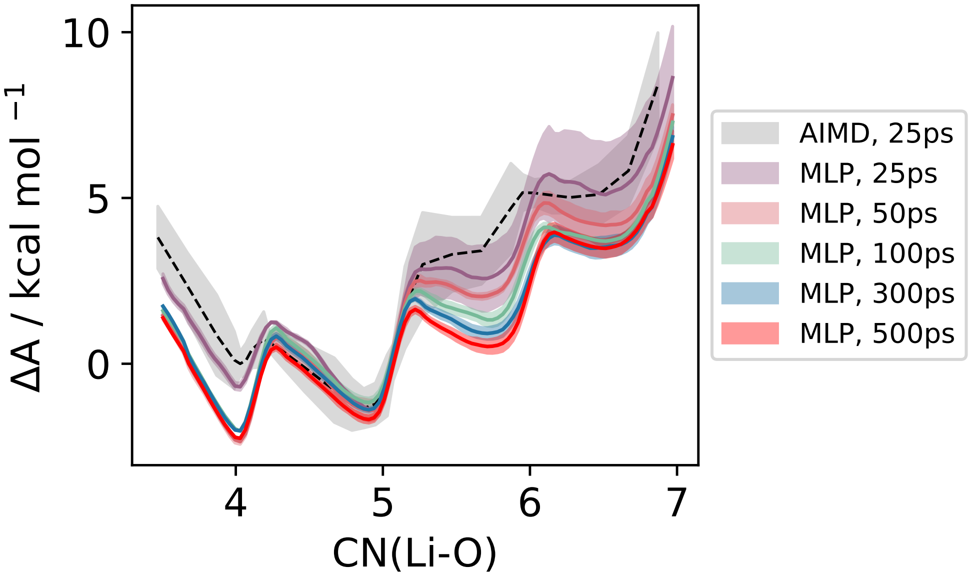

Figure S5: Free energy profile of Li_4Cl_4 computed by MLP vs AIMD.

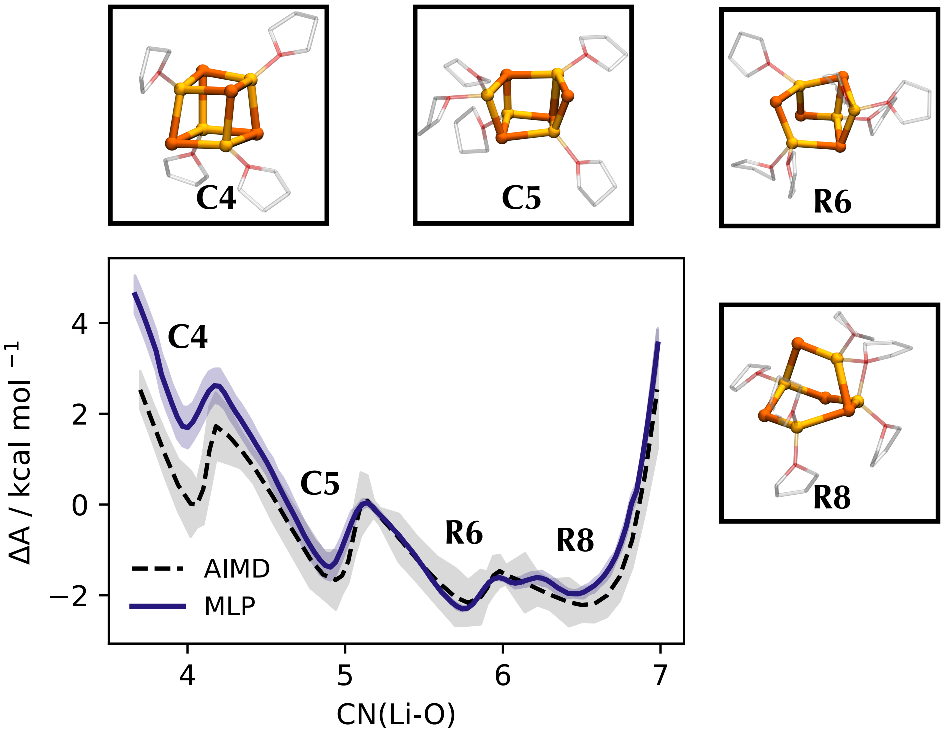

Figure S6: Free energy profile of Li_4Br_4 computed by MLP vs AIMD. Computed profiles are for 25 ps of simulations time. Li = yellow, Br = orange, C = grey, and O = red. THF hydrogens are not displayed.

The settings used for AIMD simulations are described in the previous section. Note that the cutoff was set to 250 Ry for AIMD trajectories.

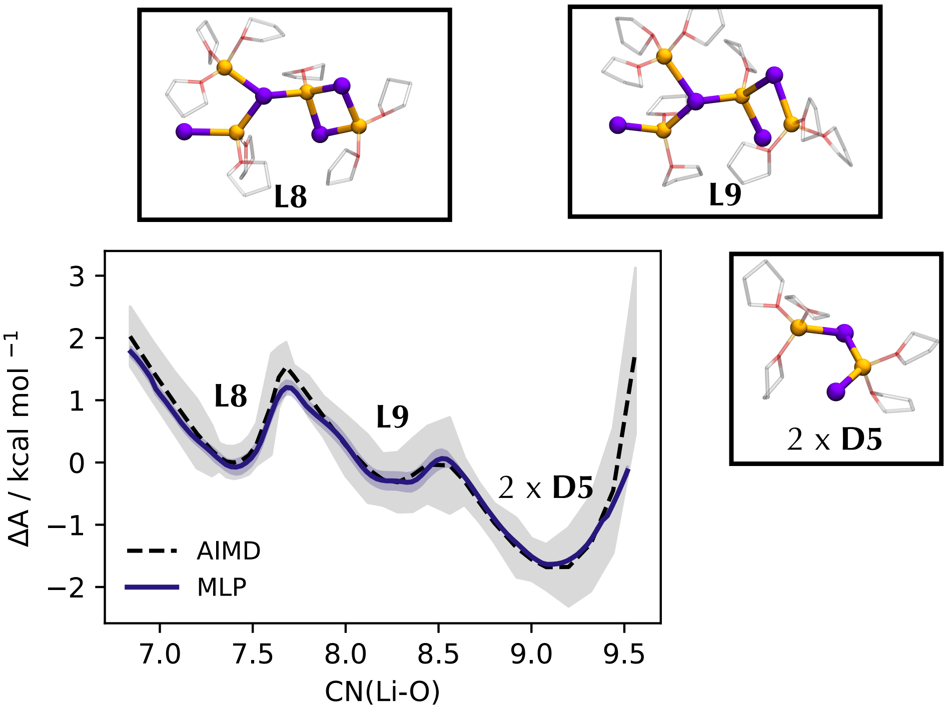

Figure S7: Free energy profile of Li_4I_4 computed by MLP vs AIMD. Computed profiles are for 25 ps of simulations time. Li = yellow, I = violet, C = grey, and O = red. THF hydrogens are not displayed.

S1.4 Production runs

The MD simulations for the production phase were carried out using LAMMPS[20] patched with the PLUMED 2 enhanced sampling plugin[21–23] and DeepMD 2[2,3]. Production runs were performed in the NVT ensemble, with a time step of 0.25 fs for the integration of the equations of motion. The temperature was kept fixed at 300 K using a CSVR algorithm[5] with 0.5 ps relaxation time. All runs were performed for a cubic lattice box in periodic boundary conditions.

A comprehensive list of the systems simulated in the production phase is provided in Table S2. Enhanced sampling techniques, comprising umbrella sampling[17] and OPES metadynamics,[24,25] were used to bias the simulations and estimate the free energy landscapes.

Table S2: Composition of the production systems and corresponding thermodynamic conditions.

System	Box side length	Temperature	Run type
Li_4Cl_4 + 55 THFs	20.1 Å	300 K	OPES
$Li_{40}Cl_{40}$ + 520 THFs	40.6 Å	300 K	umbrella sampling
Li_4Br_4 + 55 THFs	20.1 Å	300 K	OPES
Li_4I_4 + 55 THFs	20.1 Å	300 K	OPES

S1.4.1 One-dimensional solvation pathways of Li_4X_4

Umbrella sampling simulations[17] were used to reconstruct free energy profiles of Li_4X_4 by biasing the coordination number (CN) as collective variable (CV), as defined in previous works.[18,19,26]

$$\mathrm{CN(i\text{-}j)} = \frac{1-\left(\frac{\mathrm{d_{ij}}}{\mathrm{R_0}}\right)^{\mathrm{n}}}{1-\left(\frac{\mathrm{d_{ij}}}{\mathrm{R_0}}\right)^{\mathrm{m}}} \tag{S1}$$

where d_{ij} is the euclidean distance between atoms i and j, R_0 a scale parameter, and m,n free parameters. The one-dimensional free energy profiles were computed along the solvation pathway of the lithium centres:

$$\mathrm{CN(Li\text{-}O)} = \sum_{\mathrm{i}}^{\mathrm{N_{Li}}}\sum_{\mathrm{j}}^{\mathrm{N_O}}[\mathrm{CN(Li_i\text{-}O_j)}] \tag{S2}$$

where $\mathrm{CN(Li_i\text{-}O_j)}$ is the coordination number of the jth THF oxygen around the ith Li atom, with n = 12, m = 24, R_0 = 2.5 Å. We employ n = 12, m = 24, and R_0 = 3.1, 3.2, 4.0 Å for LiCl, LiBr, LiI

respectively.

The sampled potential was then reweighed using the weighted histogram method.[27,28] The block averaging method was employed to analyze the data and ensure accurate estimation of statistical uncertainties. The following settings were employed for the simulations of the three lithium halide systems:

Li_4Cl_4 42 windows with force constant 480 kcal mol^{-1} Å^{-2} in the interval CN(Li-O)=3.20-6.64 for 25, 50, 100, 300 and 500 ps simulation time. Corresponding data in Figure S5.

Li_4Br_4 25 windows with force constant 170 kcal mol^{-1} Å^{-2} in the interval CN(Li-O)=3.44-6.72 for 25 ps simulation time. Corresponding data in Figure S6.

Li_4I_4 19 windows with force constant 170 kcal mol^{-1} Å^{-2} in the interval CN(Li-O)=7.72-9.44 for 25 ps simulation time. Corresponding data in Figure S7.

S1.4.2 Higher LiCl aggregates

Umbrella sampling simulations[17] were employed to compute the binding affinities (ΔA) for higher weight LiCl, by biasing the Euclidean distance between the centre of mass (COM) of each binding unit i:

$$\mathrm{CV} = \mathrm{d}(\mathrm{COM}_1\text{-}\mathrm{COM}_2) \tag{S3}$$

For the formation of a 16 nuclei aggregate: 26 windows of force constant 1430 kcal mol^{-1} Å^{-2} in the interval d=0.3-12.1 Å for approximately 40 ps simulation time. For the formation of a 24 nuclei aggregate: 24 windows of force constant 1430 kcal mol^{-1} Å^{-2} in the interval d=4.3-12.0 Å for approximately 75 ps simulation time. Corresponding data in Figure 5. The sampled potential was then reweighed using the weighted histogram method.[27,28] The block averaging method was employed to analyze the data and ensure accurate estimation of statistical uncertainties.

S1.4.3 Two-dimensional free energy maps of Li_4X_4

The two-dimensional free energy surfaces were obtained from OPES simulations,[24,25] biasing a similarly defined collective variable as for the one-dimensional profiles in Eqs. (S1-S2):

$$\mathrm{CN}^2(\mathrm{Li\text{-}O}) = \sum_{\mathrm{i}}^{\mathrm{N_{Li}}} \left(\sum_{\mathrm{j}}^{\mathrm{N_O}} \mathrm{CN}(\mathrm{Li_i\text{-}O_j}) \right)^2 \tag{S4}$$

with parameters as follows: n = 12, m = 24, and R_0 = 3.1, 3.2, 4.0 Å for LiCl, LiBr, LiI respectively. We biased a second collective variable defined as:

$$\mathrm{CN}(\mathrm{Li\text{-}X}) = \sum_{\mathrm{i}}^{\mathrm{N_{Li}}} \sum_{\mathrm{j}}^{\mathrm{N_X}} [\mathrm{CN}(\mathrm{Li_i\text{-}X_j})] \tag{S5}$$

which defines the coordination of the Li centres to the halogen species. The OPES simulations[24,25] were run with the following settings:

Li_4Cl_4 The bias potential was updated every 500 steps, with a barrier parameter of 15.55 kcal mol^{-1} (65 kJ mol^{-1}) and simulation time of 20 ns.

Li_4Br_4 The bias potential was updated every 500 steps, with a barrier parameter of 9.57 kcal mol^{-1} (40 kJ mol^{-1}) in free energy, and simulation time of 90 ns.

Li_4I_4 The bias potential was updated every 500 steps, with a barrier parameter of 1.20 kcal mol^{-1} (5 kJ mol^{-1}) in free energy, and simulation time of 13 ns.
The barrier parameter defines the energy threshold for the free energy landscape; regions at higher free energy values are not sampled.

S2 Quantum chemical energies of higher weight LiCl

The single point energies for snapshots of the higher weight aggregates of LiCl were also computed at the DFT/PBE, 6-31+g(d,p) level using implicit THF as IEFPCM and explicit THF for the first coordination shell of Li; for limits in the wavefunction convergence, this was not included for $Li_{12}Cl_{12}$.

Dispersion correction was included as D3 empirical dispersion.[15] The single point energies are given in Table S3. The formation of Li_4Cl_4 + Li_4Cl_4 →Li_8Cl_8 is accompanied by a release of 13 and 20 kcal mol^{-1} of energy for IEFPCM and IEFPCM+explicit, respectively. The further aggregation of Li_8Cl_8+Li_4Cl_4→$Li_{12}Cl_{12}$ is favoured by 13 kcal mol^{-1}, for IEFPCM.

Table S3: Single point energies of various LiCl aggregates computed at the DFT/PBE level, and stabilisation energies (Δ) for the formation of such aggregates, from single point and MLP calculations. Δ_1 refers to the formation of a 16-nuclei aggregate from the combination of 2 8-nuclei aggregates, and Δ_2 refers to its incorporation with another 8-nuclei aggregate. Single point energies in Hartrees, and stabilisation energies in kcal mol^{-1}

	Li_4Cl_4	Li_8Cl_8	$Li_{12}Cl_{12}$	Δ_1	Δ_2
DFT/PBE + IEFPCM	-1870.54	-3741.10	-5611.66	-13	-13
DFT/PBE + IEFPCM + explicit	-2799.23	-5598.50	–	-21	–
MLP				-16.1(8)	-10(1)

S3 Atomic charges

CM5 charges[29] were calculated using the Gaussian software package[30] on representative monomeric, dimeric, and cubane aggregates for the three lithium halides (Figure S8). The choice of the CM5 charges is due to their improved performance in describing dipole moments.[29]

The calculations were run at the DFT/PBE level,[10,11] with D3 empirical dispersion[15], and 6-31+G(d,p) basis set[31–33] for X = Cl, Br and 6-311G(d,p)[34–37] for X = I. Explicit THF solvent was used for the first coordination shell, and implicit modeling of THF for the outer shells, using the integral equation formalism variant of the polarizable continuum model (IEFPCM).[38–40] Reported charges represent averages for snapshots taken every 500 fs of a 20 ps NVT run at 300 K. For LiI, we additionally computed atomic charges in the gas phase, as shown in Table S4.

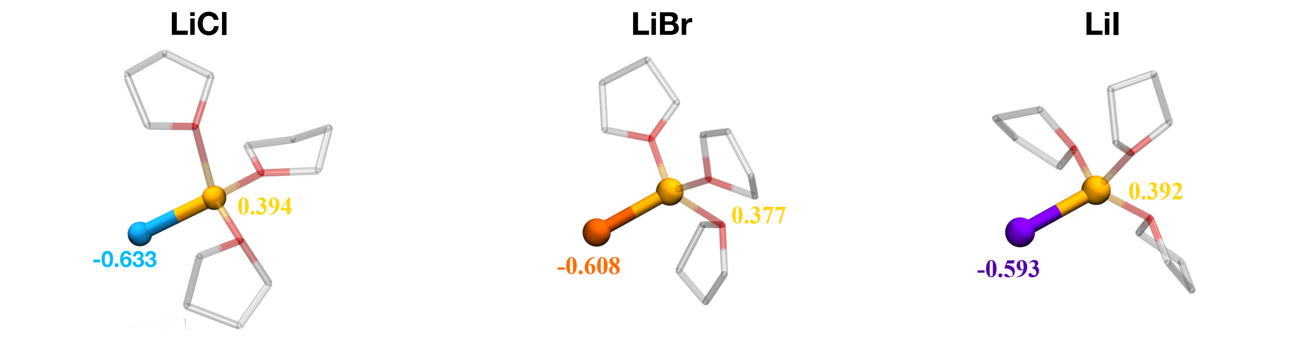

(a) Monomer.

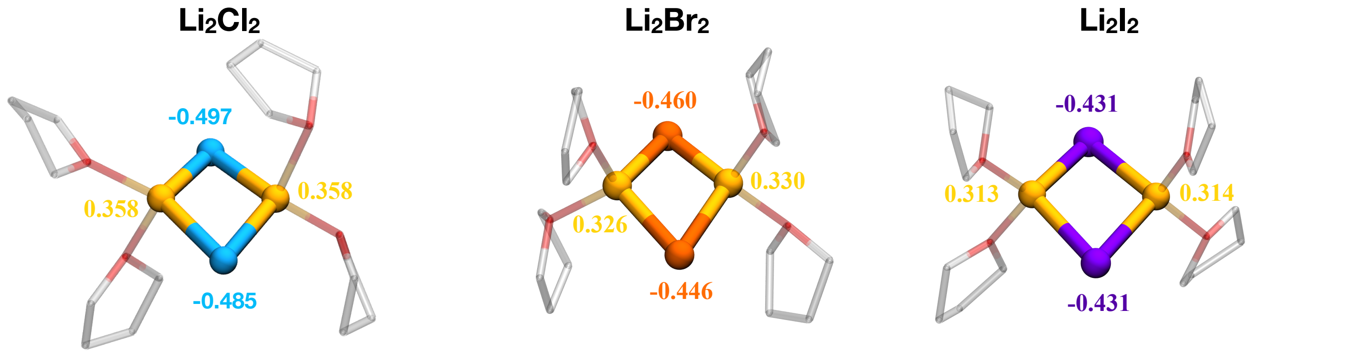

(b) Dimer.

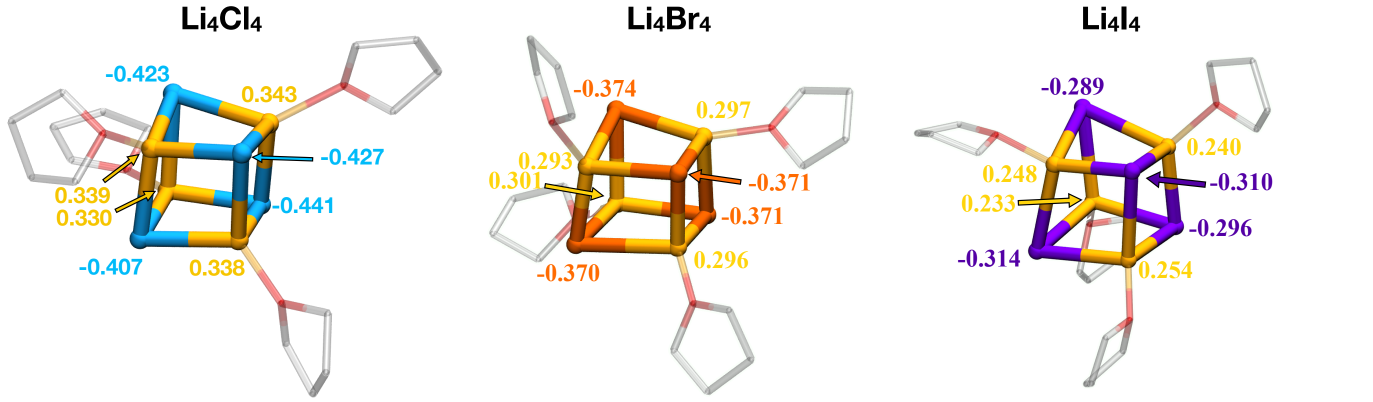

(c) **C4** tetramer.

Figure S8: Charges of different sized aggregates of the three lithium halides considered in this study. Li = yellow, Cl = cyan, Br = orange, I = violet, C = grey, O = red. THF hydrogens are not displayed.

Table S4: Atomic charges for Li, I, and charge differences (Δ) for various LiI aggregates in the gas phase and in THF solution. Li = yellow, I = violet, C = grey, and O = red. THF hydrogens are not displayed.

	**Gas phase**			**In THF**		
	LiI	Li_2I_2	Li_4I_4	$LiI(THF)_3$	$Li_2I_2(THF)_4$	$Li_4I_4(THF)_4$
	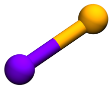	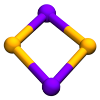	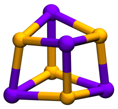	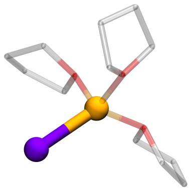	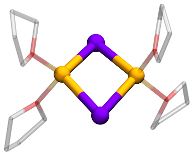	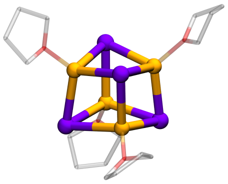
Li	0.651	0.468	0.333	0.392	0.314	0.244
I	-0.651	-0.468	-0.333	-0.593	-0.425	-0.302
Δ	1.302	0.936	0.666	0.985	0.739	0.546

S4 Probability distribution function for Li-THF

The probability distribution function for the average number of THF per Li was computed over 5 ns of unbiased NVT run. For all systems, the initial configuration corresponded to a pseudo-cubane **C4**. The average number of THF surrounding the Li atoms was computed from the total Li-O coordination number as described in Equation S2.

S5 Halogen-THF affinity

Halogen-THF affinities were estimated from the radial pair distribution functions (RDFs) over X-C_{THF} pairs computed on NVT runs of 1 unit of monomeric LiX species in THF as $\Delta A = \Delta V \cdot N$, where ΔA is the total binding affinity between X-C_{THF}, ΔV is the binding affinity per C atom computed from the Potential of Mean Force (PMF), and N is the average number of C neighbors (Figure S9). The THFs belonging to the first coordination shell of Li were excluded from the computation of the RDF.

Table S5: Thermodynamic parameters for halogen-THF interactions computed on LiX monomers in THF. Binding affinities are given in kcal mol^{-1}.

X	ΔV	N	ΔA
Cl	-0.20(9)	35	7(3)
Br	-0.28(6)	50	14(3)
I	-0.30(6)	60	18(4)

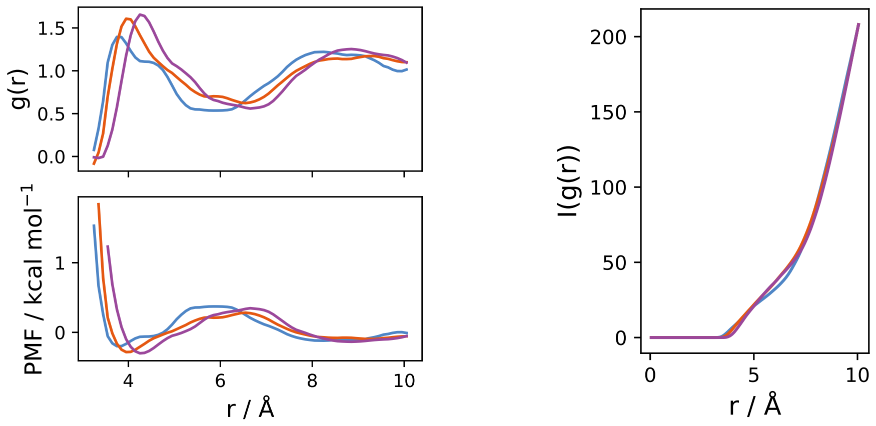

Figure S9: Radial pair distribution function (g(r)) (left, top), potential of mean force (PMF) (left, bottom), and number integral I(g(r)) (right) between non hydrogen THF atoms and the halogen, for LiX salt solutions. Blue lines: X = Cl, Orange lines: X = Br, Purple lines: X = I.

References

(1) L. Zhang, J. Han, H. Wang, W. A. Saidi, R. Car and W. E, *arXiv preprint arXiv:1805.09003v2*, 2018.

(2) H. Wang, L. Zhang, J. Han and W. E, *Comput. Phys. Commun.*, 2018, **228**, 178–184.

(3) J. Zeng *et al.*, *J. Chem. Phys.*, 2023, **159**, 054801.

(4) W. G. Hoover, *Phys. Rev. A*, 1986, **34**, 2499–2500.

(5) G. Bussi, D. Donadio and M. Parrinello, *J. Chem. Phys.*, 2007, **126**, .

(6) M. Yang, L. Bonati, D. Polino and M. Parrinello, *Catal. Today*, 2022, **387**, 143–149.

(7) S. Vandenhaute, M. Cools-Ceuppens, S. DeKeyser, T. Verstraelen and V. V. Speybroeck, *Npj Comput. Mater.*, 2023, **9**, 1–8.

(8) P. Hohenberg and W. Kohn, *Phys. Rev.*, 1964, **136**, B864–B871.

(9) W. Kohn and L. J. Sham, *Phys. Rev.*, 1965, **140**, A1133–A1138.

(10) J. P. Perdew, K. Burke and M. Ernzerhof, *Phys. Rev. Lett.*, 1996, **77**, 3865–3868.

(11) J. P. Perdew, K. Burke and M. Ernzerhof, *Phys. Rev. Lett.*, 1997, **78**, 1396–1396.

(12) G. Lippert, J. Hutter and M. Parrinello, *Theor. Chem. Acc.*, 1999, **103**, 124–140.

(13) J. VandeVondele and J. Hutter, *J. Chem. Phys.*, 2007, **127**, 114105.

(14) S. Goedecker, M. Teter and J. Hutter, *Phys. Rev. B*, 1996, **54**, 1703–1710.

(15) S. Grimme, J. Antony, S. Ehrlich and H. Krieg, *J. Chem. Phys.*, 2010, **132**, 154104.

(16) T. D. Kühne *et al.*, *J. Chem. Phys.*, 2020, **152**, 194103.

(17) G. Torrie and J. Valleau, *J. Comput. Phys.*, 1977, **23**, 187–199.

(18) M. Iannuzzi, A. Laio and M. Parrinello, *Phys. Rev. Lett.*, 2003, **90**, 238302.

(19) M. de Giovanetti, S. H. Hopen Eliasson, A. C. Castro, O. Eisenstein and M. Cascella, *J. Am. Chem. Soc.*, 2023, **145**, 16305–16309.

(20) A. P. Thompson, H. M. Aktulga, R. Berger, D. S. Bolintineanu, W. M. Brown, P. S. Crozier, P. J. in 't Veld, A. Kohlmeyer, S. G. Moore, T. D. Nguyen, R. Shan, M. J. Stevens, J. Tranchida, C. Trott and S. J. Plimpton, *Comput. Phys. Commun.*, 2022, **271**, 108171.

(21) M. Bonomi, D. Branduardi, G. Bussi, C. Camilloni, D. Provasi, P. Raiteri, D. Donadio, F. Marinelli, F. Pietrucci, R. A. Broglia and M. Parrinello, *Comput. Phys. Commun.*, 2009, **180**, 1961–1972.

(22) G. A. Tribello, M. Bonomi, D. Branduardi, C. Camilloni and G. Bussi, *Comput. Phys. Commun.*, 2014, **185**, 604–613.

(23) M. Bonomi *et al.*, *Nat. Methods*, 2019, **16**, 670–673.

(24) M. Invernizzi and M. Parrinello, *J. Phys. Chem. Lett.*, 2020, **11**, 2731–2736.

(25) M. Invernizzi and M. Parrinello, *J. Phys. Chem. Lett.*, 2021, **12**, 912–912.

(26) R. Peltzer, O. Eisenstein, A. Nova and M. Cascella, *J. Phys. Chem. B*, 2017, **121**, 4226–4237.

(27) A. Grossfield, *WHAM: the weighted histogram analysis method*, Version 2.0.10, `http://membrane.urmc.rochester.edu/wordpress/?page_id=126`.

(28) S. Kumar, J. M. Rosenberg, D. Bouzida, R. H. Swendsen and P. A. Kollman, *J. Comput. Chem.*, 1992, **13**, 1011–1021.

(29) A. V. Marenich, S. V. Jerome, C. J. Cramer and D. G. Truhlar, *J. Chem. Theory Comput.*, 2012, **8**, 527–541.

(30) M. J. Frisch *et al.*, *Gaussian 16 Revision C.01*, 2016.

(31) M. M. Francl, W. J. Pietro, W. J. Hehre, J. S. Binkley, M. S. Gordon, D. J. DeFrees and J. A. Pople, *J. Chem. Phys.*, 1982, **77**, 3654–3665.

(32) M. S. Gordon, J. S. Binkley, J. A. Pople, W. J. Pietro and W. J. Hehre, *J. Am. Chem. Soc.*, 1982, **104**, 2797–2803.

(33) G. W. Spitznagel, T. Clark, P. v. R. Schleyer and W. J. Hehre, *J. Comput. Chem.*, 1987, **8**, 1109–1116.

(34) M. N. Glukhovtsev, A. Pross, M. P. McGrath and L. Radom, *J. Chem. Phys.*, 1995, **103**, 1878–1885.

(35) B. P. Pritchard, D. Altarawy, B. Didier, T. D. Gibsom and T. L. Windus, *J. Chem. Inf. Model.*, 2019, **59**, 4814–4820.

(36) D. Feller, *J. Comput. Chem.*, 1996, **17**, 1571–1586.

(37) K. L. Schuchardt, B. T. Didier, T. Elsethagen, L. Sun, V. Gurumoorthi, J. Chase, J. Li and T. L. Windus, *J. Chem. Inf. Model.*, 2007, **47**, 1045–1052.

(38) S. Miertuš, E. Scrocco and J. Tomasi, *Chem. Phys.*, 1981, **55**, 117–129.

(39) S. Miertuš and J. Tomasi, *Chem. Phys.*, 1982, **65**, 239–245.

(40) J. L. Pascual-ahuir, E. Silla and I. Tuñon, *J. Comput. Chem.*, 1994, **15**, 1127–1138.
